# The development of a capacity-strengthening program to promote self-care practices among people with lymphatic filariasis-related lymphedema in the Upper West Region of Ghana

**DOI:** 10.1186/s40249-021-00846-z

**Published:** 2021-05-07

**Authors:** Solomon Abotiba Atinbire, Benjamin Marfo, Bright Alomatu, Collins Ahorlu, Paul Saunderson, Stefanie Weiland

**Affiliations:** 1 Accelerating Integrated Management (AIM) Initiative, 27 Jungle Road, Accra, Ghana; 2 National NTD Programme, Accra, Ghana; 3 Noguchi Memorial Institute for Medical Research, University of Ghana, Accra, Ghana; 4 AIM Initiative-American Leprosy Missions, Greenville, SC USA

**Keywords:** Lymphedema, Self-care, Training, Ghana

## Abstract

**Background:**

The Upper West region of Ghana is mostly made up of rural communities and is highly endemic for lymphatic filariasis (LF), with a significant burden of disability due to lymphedema and hydrocele. The aim of this paper is to describe an enhanced, evidence-based cascading training program for integrated lymphedema management in this region, and to present some initial outcomes.

**Main text:**

A baseline evaluation in the Upper West Region was carried out in 2019. A cascaded training program was designed and implemented, followed by a roll-out of self-care activities in all 72 sub-districts of the Upper West Region. A post implementation evaluation in 2020 showed that patients practiced self-care more frequently and with more correct techniques than before the training program; they were supported in this by health staff and family members.

**Conclusions:**

Self-care for lymphedema is feasible and a program of short workshops in this cascaded training program led to significant improvements. Efforts to maintain momentum and sustain what has been achieved so far, will include regular training and supervision to improve coverage, the provision of adequate resources for limb care at home, and the maintenance of district registers of lymphedema cases, which must be updated regularly.

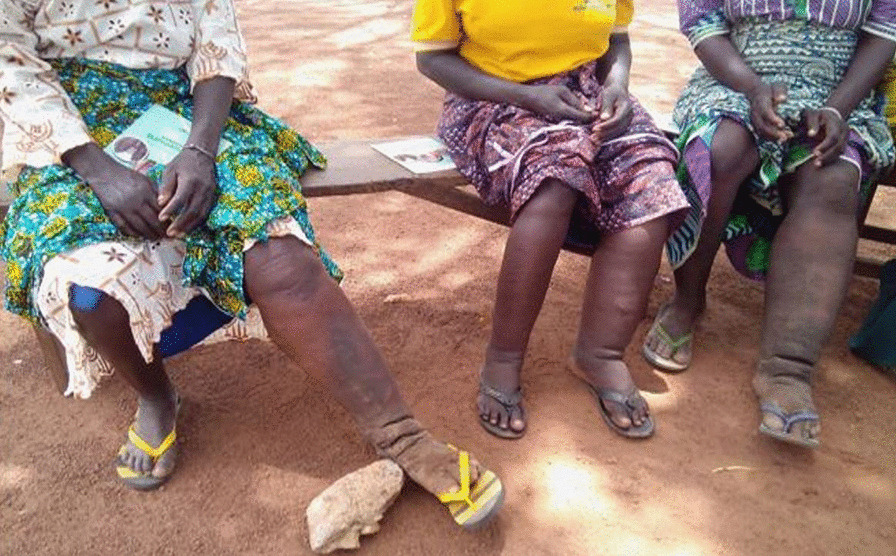

## Background

Lymphatic filariasis (LF) is one of the most widespread neglected tropical diseases (NTDs), and the suffering caused by the disease sequelae (in particular, lymphedema and hydrocele) is enormous. It has been previously estimated that about 14 million people suffer from lymphedema worldwide (73 countries are regarded as endemic), and the condition is made worse by frequent attacks of acute bacterial dermatolymphangioadenitis (ADLA) [1]. A 2019 paper on registered cases reported to the World Health Organization (WHO), gives a figure of 1.1 million lymphedema cases due to LF worldwide, but this probably misses many unreported cases, especially mild cases [2]. While the true number may have declined from the figure of 14 million in 1996 as the disease is gradually being eliminated, the consequences of LF are generally lifelong; the number reported by WHO has increased slightly in recent years, as registration of cases improves.

The Global Programme to Eliminate Lymphatic Filariasis (GPELF), launched by the WHO in 2000, has conducted annual campaigns of mass drug administration (MDA) in endemic areas, which have brought about a significant decrease in disease transmission. Countries involved in the programme were required to deliver preventive chemotherapy and to provide a basic package of care for people with existing morbidity (lymphedema and hydrocele) [3]. A systematic review and meta-analysis of hygiene-based interventions published in 2015 noted that scaling up of morbidity management activities has been slow, partly because of the lack of proven, evidence-based interventions [4]. A study on the economic costs and benefits of community-based lymphedema management in India, however, concludes that such interventions are highly cost-effective, with per-person savings of 185 times the program’s per-person costs [5].

Chronic lymphedema is a life-long condition and poverty is common in areas endemic for LF, with poor access to health services. In these circumstances, most of the burden for care falls on patients themselves and their families, through self-care strategies. Recent reviews have proposed a self-care protocol for people affected by moderate to severe lymphedema [6, 7]. This protocol covers several areas such as:

- Hygiene; washing and drying, attending to skin lesions, medicated cream and trimming nails
- Exercise; standing, seated and lying exercises, day- and night-time elevation, walking, deep breathing
- Massage; mobilizing skin and tissues, lymphatic massage
- Management of acute attacks and accessing referral services

Interventions needed to manage disease-related morbidity in resource-poor settings have been known for decades, but a greater focus on self-care has only emerged relatively recently [8, 8]. In an innovative programme for both leprosy and lymphedema due to LF in southern Nepal, good self-care was found to lead to reduced stigma, with a key feature being an increase in self-respect and a feeling of empowerment in those involved, leading to better self-care and further empowerment [10, 11]. Issues of personal attitude and motivation have been noted as important for self-care in other contexts, such as psoriasis [12].

Ghana has made significant progress in reducing the transmission of LF. According to national NTD program data, by 2020, 99 of 114 endemic districts had achieved interruption of transmission, confirmed through transmission assessment surveys, and have thus stopped MDA. However, the provision of morbidity management and disability prevention (MMDP) services has not been as successful, even though the LF control program takes advantage of the annual MDAs to collect information on the number of suspected hydrocele and lymphedema cases in endemic communities/districts. Mapping of LF morbidity using data from community drug distributors (CDD) was carried out in 2015, clearly illustrating the very high density of cases in the northern regions [13]. Thus, about 5000 cases of lymphedema and 10 000 cases of hydrocele have been registered in the country, according to an NTD working paper from the Ghana Health Service, many of which have gone unattended due to financial constraints; additionally, many suspected cases are not confirmed by health professionals and remain hidden due to stigma, lack of awareness and the limited capacity of the health system to respond. The national NTD program estimates that there are almost 700 cases of lymphedema in the 11 districts (72 sub-districts) of the Upper West Region.

In 2016, the NTD Programme developed a package of care for MMDP that relied primarily on a cascaded training approach to improve capacity within the health system. After a pilot phase, the current programme for the Upper West Region was proposed. This paper reports on the development and implementation of a comprehensive package of training in the Upper West Region, covering all districts and each level of health staff, down to the community-based volunteers and family members. This project was carried out in the context of parallel work on preparation of an LF elimination dossier for Ghana.

## Methods

### Project setting

The aim of the project was to develop and implement a cascaded training program to enable better access to MMDP services for lymphatic filariasis patients suffering from lymphedema and adenolymphangitis. It was implemented in all 11 districts of the Upper West Region of Ghana. The region is among twelve endemic regions for LF, with the highest burden of lymphedema cases in the country. Of the eleven districts in the region that were included in the MDA distribution, eight have interrupted transmission and are now focused on providing MMDP services to people affected by LF.

### Description of the cascaded capacity-building intervention

The cascaded capacity-building intervention was preceded by a formative evaluation to identify capacity gaps and needs among health supervisors, frontline providers, patient support groups, CDDs and patients themselves. The training participants included purposely selected respondents who were currently or previously involved in MMDP service provision, as well as patients, community leaders or patients’ support groups in the study region.

Following the needs evaluation, training materials were developed to address the gaps in capacity to provide MMDP services to patients by care providers, and build patients’ ability and that of their support groups to self-manage lymphedema at home. Three cascaded training workshops were organized to train service providers on MMDP integration, self-care, monitoring, supervision and reporting of MMDP activities. The first workshop focused on orienting regional-level supervisors to rollout the capacity-building cascaded training; the second workshop was for district-level managers and service providers, and the third was a step-down workshop for frontline health providers, patients themselves, patients’ support groups, family members and CDDs.

The formation of self-care groups in every district of the region with lymphedema patients, was considered a critical first step for proper lymphedema management at home to increase the general well-being of their members.

In the first and second training workshops, providers and supervisors were trained together, whereas parallel sessions of cascaded training were adopted for the third workshop in each district. Each workshop lasted for one day. Health facility staff and other caregivers were trained to provide a minimum package of care to patients, which focused on hygiene, treatment of acute attacks, and managing lymphedema; managing hydrocele and LF treatment were discussed, but are not part of the self-care package. A key goal was to improve access to care by bringing services to the doorstep of patients. The training took place between September and December 2019.

### Subsequent field work

Following completion of the training workshops, management of lymphedema has been taking place in the home and in the peripheral health facilities. These activities were examined during monitoring visits. An evaluation in mid-2020 looked at the outcomes in terms of increased care and reduced morbidity.

### Data collection and analysis

Various data collection tools were used; these included in-depth interviews (IDIs) and a questionnaire survey. Qualitative data were analyzed using an iterative process, beginning during data collection through daily debriefings and review of data and emergent themes. MaxQda 2020 (Verbi GmbH, Berlin, Germany)—a qualitative data analysis software program for storage, indexing, and retrieval—was used to thematically analyze the IDI transcripts, selecting the most representative quotations for reporting such that no minority or majority view was suppressed. The survey data were analyzed using EpiInfo 3.5.4 (Centers for Disease Control and Prevention, Atlanta, USA) package to generate frequencies for reporting. Comparison of frequencies was carried out using the Chi square test, with significance determined by *P* < 0.05.

## Results and discussion

### Cascaded capacity-building workshops

The development of a training program to improve the management of lymphedema in northern Ghana aimed at building the capacity of health workers to provide care for patients, reducing stigma in the community, and promoting MMDP integration into regular training programs. The NTD Programme developed the training content based on the WHO capacity-building training manual [14]. Table 1 describes the features of the training program in Upper West Region, while Table 2 shows in more detail the participation in the various training workshops.

**Table 1:** Features of the capacity-building interventions Upper West Region of Ghana

Conducted 2019–2020
Covered all 11 districts in Upper West Region
Included all levels of health staff, down to Community Health Planning (CHP) services facilities, community volunteers and family members
Covered regional health management team, staff from regional hospital, district hospitals, health centers/clinics and each CHP across all 72 sub-districts
Included tutors from health professional training institutions (nursing and midwifery training colleges)
Relied primarily on a cascaded training approach
Provided essential supplies (consumables) to health facilities and to affected persons trained in self-care to carry out self-care at home
Involved the formation of self-care groups in every district that would come to health facilities for health education days
Training included morbidity management and disability prevention (MMDP) content, patient presentation and demonstration, plus monitoring, supervision, and reporting of MMDP activities tailored to management and regional/district staff
Training manuals provided. Also, registers for documenting services were printed and provided for each level of the health system down to the health-facility level
A system was created for reporting data recorded in registers to the national level through neglected tropical diseases coordinators and health information offices
Every person affected attended training with a family support person, who was also trained in managing self-care, assisting with seeking care, and reducing stigma

**Table 2:** Cascaded capacity-building workshops attended by participants in Upper West; numbers attending (%)

Training workshops organized by the NTD Programme	Topics covered	Service providers and supervisors (*n* = 506)	Patients (*n* = 293)	Family support group (*n* = 302)	CDDs (*n* = 598)
Workshop 1: Training of trainer for regional-level managers	Monitoring, supervision and reporting of MMDP activities Assessment and staging of lymphoedema Integration of MMDP into routine activities in districts and the region Washing of affected part Exercising of affected part Limb elevation Proper wound care Prescription of appropriate footwear	14 (2.8%)	–	–	–
Workshop 2: Training of trainers of district-level service supervisors	Integration of MMDP into routine activities in districts and the region Monitoring, supervision and reporting of MMDP activities Assessment and staging of lymphoedema Washing of affected part Exercising of affected part Limb elevation Proper wound care Prescription of appropriate footwear	160 (31.6%)	60 (20.5%)	52 (17.2%)	–
Step-down training on MMDP for frontline service providers and users	Assessment and staging of lymphoedema Washing of affected part Exercising of affected part Limb elevation Proper wound care Prescription of appropriate footwear	332 (65.6%)	233 (79.5%)	250 (82.8%)	598 (100%)

*CDDs* community drug distributors, *MMDP* morbidity management and disability prevention, *NTDs* neglected tropical diseases, – not applicable

### Regional-level orientation

A four-member regional-level core MMDP technical team was constituted to lead and provide technical support to the district and sub-district level teams. This was made up of the regional NTD focal person and three other disease control and surveillance officers. The regional team was given a one-day training on lymphedema management to equip them with the required skills to train districts and also be able to manage cases on their own. The training included demonstration sessions with patients.

### District-level training

Four training teams were formed, with membership from the national facilitators and the trained regional core team, to facilitate the trainings in the districts. From the sub-districts, a clinician and a public health staff from the main health facility were invited. All sub-district level facilities presented a case at the training for demonstration in lymphedema management. Six nursing and midwifery training colleges in the region also participated in the training, as part of a long-term goal of getting LF MMDP included in the curricula of health training institutions.

### Sub-district level training

The sub-district level training was done for staff at Community Health Planning (CHP) compounds and CDDs with the aim of bringing services closer to patients. CDDs were trained on case identification and mobilization of patients to access care as well as to provide support for patients in the communities. The trainings were facilitated by the sub-district teams and supported by the district and regional teams. All 72 sub-districts carried out the training in December 2019.

Monthly follow-up supervision and monitoring visits were done after the training workshops. When training participants were assessed during the supervisory visits, they demonstrated knowledge on LF MMDP care by answering questions posed to them satisfactorily.

In order to improve access, health workers at community level were included in the training. To support self-care at home, a family member was trained to understand the disease and the needs of the patient. New patients are encouraged to bring a family member who can encourage and provide support in self-care. CDDs from communities with cases were trained to provide support to the patients and also provide the linkage between the patients and the peripheral health workers.

### End evaluation

One hundred and sixty-four lymphedema patients in the Upper West Region were interviewed using a structured questionnaire on patient hygiene behavior at home and lymphedema morbidity management and prevention techniques. This was done approximately six months after the training workshops were held. It was possible to compare many of the results with the findings of the baseline survey carried out one year previously in the same region with 173 participants. Respondents in the two surveys were similar in terms of demographic and socio-economic indicators. Both surveys were carried out by one of the authors.

### Home management of lymphedema

A majority of the LF patients interviewed during the evaluation reportedly wash their affected limbs with soap and water in a specific manner, either by themselves or with the assistance of someone at home. LF patients indicated that the MMDP intervention implemented by the program has enabled them to adopt appropriate hygiene behavior. For instance, the recommended practice of washing the limbs at least once or more daily was practiced by LF patients with about 51% of them washing their affected limbs more than once per day (Table 3).

**Table 3:** Frequency of washing affected limb with soap and water in the advised manner, at baseline and at the end evaluation

Variables	Baseline (*n* = 173)	Evaluation (*n* = 164)	*P* value
	*n* (frequency, %)	*n* (frequency, %)	
More than once per day	55 (31.8)	83 (50.6)	0.001
Once daily	31 (17.9)	48 (29.3)	= 0.019
More than once per week	16 (9.2)	6 (3.7)	= 0.063
Once per week	11 (6.4)	11 (6.7)	= 0.928
More than once per month	15 (8.7)	1 (0.6)	= 0.001
Once per month	45 (26.0)	15 (9.1)	< 0.001

Various lymphedema morbidity management and prevention techniques were reported by respondents as shown in Table 4, with an increase in recommended practices and a decrease in the use of traditional remedies. There were also changes in the strategies used to manage acute attacks at home, as shown in Table 5.

**Table 4:** Lymphedema morbidity prevention/management techniques among lymphatic filariasis patients, at baseline and at the end evaluation

Variables	Baseline (*n* = 173)	Evaluation (*n* = 164)	*P* value
	*n* (frequency, %)	*n* (frequency, %)	
Hygiene/washing and drying the affected limbs	57 (32.9)	120 (73.2)	< 0.001
Wound care/care for lesions	43 (24.9)	84 (51.2)	< 0.001
Elevation of affected limbs	20 (11.6)	70 (42.7)	< 0.001
Exercise	15 (8.7)	53 (32.3)	< 0.001
Prophylactic creams	16 (9.2)	37 (22.6)	= 0.001
Prophylactic systemic antibiotics	46 (26.6)	16 (9.8)	< 0.001
Use of shoes/sandals	8 (4.6)	10 (6.1)	= 0.718
Traditional remedies	37 (21.4)	3 (1.8)	< 0.001
Other (no stress, less work, etc.)	0 (0.0)	2 (1.2)	= 0.455
Don’t know any prevention means	58 (33.5)	0 (0.0)	< 0.001

Multiple choices allowed. Sorted on column 3 in descending order

**Table 5:** Treatment strategies for acute attacks, at baseline and at the end evaluation

Variables	Baseline (*n* = 173)	Evaluation (*n* = 164)	*P* value
	*n* (frequency, %)	*n* (frequency, %)	
Cool affected limb in cold water/cold compress	41 (23.7)	115 (70.5)	< 0.001
Visiting the health facility	87 (50.3)	82 (50.0)	> 0.05
Having enough rest	48 (27.7)	67 (40.8)	= 0.015
Elevation of the affected limb	25 (14.4)	52 (31.7)	< 0.001
Drink more fluid	20 (11.6)	37 (22.6)	= 0.011
Apply antibiotics on affected skin	36 (20.8)	16 (9.8)	= 0.007
Avoid exercise during acute attacks	12 (6.9)	10 (6.1)	> 0.05
Take antibiotics orally	73 (42.2)	2 (1.2)	< 0.001
Inject antibiotics	9 (5.2)	1 (0.6)	= 0.031
Traditional remedies	38 (10.9)	0 (0.0)	< 0.001
Visiting traditional healers	27 (15.6)	0 (0.0)	< 0.001
Don’t know any acute attack treatment	17 (9.8)	0 (0.0)	< 0.001

Multiple choices allowed. Sorted on column 3 in descending order

In terms of morbidity, almost all patients with lymphedema continued to experience occasional signs of an acute attack, however, the frequency of the attacks has reduced significantly: at baseline, 90 (54%) of the patients experienced these symptoms more than twice per year, while at evaluation, only 54 (34%) reported same (*P* < 0.001).

The views and attitudes of community leaders and health staff were also sought during the evaluation. In general, the training was credited with providing knowledge about the disease, which could lead to improved care and reduced stigma. Another theme derived from the interviews was the problem of poverty and lack of resources to facilitate health seeking among patients. Patients have difficulty in accessing the health services, while health staff are unable to do as many home visits as they would like. Supplies for limb and wound care are often unavailable.

Many challenges, including the need for resources to support the peripheral health services, remain to be addressed:

- Follow-up visits are often not happening due to fuel constraints and the coronavirus disease 2019 pandemic. This has also adversely affected the formation and functioning of self-care groups.
- Although all districts and sub-districts were covered, resource constraints prevented the registration and involvement of many patients in remote areas, so overall coverage was less than 50%.
- It came to light that not many patients with lower grades of lymphedema who could have benefited from the morbidity management training were being included. The additional value of inviting them includes helping them to be aware of the importance of leg hygiene in controlling acute attacks and slowing down disease progression.
- There are still many more staff at the peripheral health facilities and at the community level, who need training, as well as many patients, so an ongoing program of training is needed, also bearing in mind a certain level of staff turnover.

Patients suffering from the consequences of LF are poorly served in most endemic areas. They frequently suffer from stigmatization and discrimination, and live in poverty [15, 15]. This study also found that local beliefs about the causes of these conditions were common, often leading patients to rely on traditional healers as the first choice of care providers.

This study has shown, however, that even in remote and impoverished areas it is not impossible to change attitudes and that simple techniques of self-care can have a significant effect. The frequency of washing the affected limb increased significantly after the training, while helpful practices (such as elevating the affected limb, or cooling the affected limb in an acute attack) were used more frequently and potentially harmful practices (such as traditional remedies) less frequently.

One criticism of cascaded training is that quality may be diluted as one moves down the ladder. To mitigate these negative effects, the national team and regional officers supported the district level training, and also regional officers supported the district teams in training of community workers at the sub-district level, for maximum impact. In addition, this training program was very brief and focused, taking place within the space of less than four weeks, which may have helped to maintain quality.

The perception of participants (including health staff, patients and family members) regarding the importance of leg hygiene changed after the MMDP training. The training of the family support groups has been hugely appreciated by family members.

The main limitations of the study are its short-term duration and the lack of resources to improve the capacity of peripheral health staff to visit patients more frequently and provide the supplies they may need. There is an ongoing discussion as to whether nationwide insurance cover for the required supplies can be approved, which would be a very helpful long-term solution.

## Recommendations

- A sustainable, regular training program at peripheral levels.
- The training modules should be reviewed and updated regularly.
- Milder grades of lymphedema should be included.
- Ensure availability of supplies for limb and wound care.
- Registers should be available and used in all peripheral health facilities.
- Regular supervision of lymphedema management, whether at home or health facility.
- Formation of self-care groups for health education and mutual support.
- Possible use of electronic registers.
- Integration with other chronic conditions requiring self-care, such as leprosy and Buruli ulcer.

The NTD Programme has advocated for the scale up of this enhanced capacity-building intervention to increase access to lymphedema management and care in remaining LF-endemic districts across the country. Based on the results of this study, scaling up could be of significant benefit to people affected by lymphedema by empowering them to care for themselves, which may lead to improved disease outcomes, reduced stigma related to their condition, and well-equipped family members and lower-level health system workers to care for the patients.

## Data Availability

The data behind this article can be obtained from the author on request.

## Abbreviations

ADLA: Acute bacterial dermatolymphangioadenitis
CDD: Community drug distributors
CHP: Community Health Planning
GPELF: Global Programme to Eliminate Lymphatic Filariasis
IDIs: In-depth interviews
LF: Lymphatic filariasis
MDA: Mass drug administration
MMDP: Morbidity management and disability prevention
NTDs: Neglected tropical diseases
WHO: World Health Organization
